# Silver Negative Pressure Dressing With Vacuum-assisted Closure of Massive Pelvic and Extremity Wounds

**DOI:** 10.1007/s11999-013-3123-3

**Published:** 2013-06-29

**Authors:** Herrick J. Siegel, Diego F. Herrera, Jason Gay

**Affiliations:** 1 Orthopaedic Oncology, Department of Surgery, UAB School of Medicine, Birmingham, AL 35305 USA; 2 Department of Surgery, UAB School of Medicine, Birmingham, AL USA; 3 Orthopaedic RNFA, UAB Medical Center, Birmingham, AL USA

## Abstract

**Background:**

Massive soft tissue loss involving the pelvis and extremities from trauma, infections, and tumors remains a challenging and debilitating problem. Although vacuum-assisted closure (VAC) technology is effective in the management of soft tissue loss, the adjunct of a silver dressing in the setting of massive wounds has not been as well tested.

**Questions/purposes:**

Does a silver negative pressure dressing used in conjunction with a wound VAC decrease (1) the length of acute hospital stay and overall length of treatment; (2) the number of surgical débridements the patients underwent as part of their care; and (3) the likelihood of wound closure without soft tissue transposition?

**Methods:**

We evaluated 42 patients with massive (> 200 cm^2^) pelvic and extremity wounds from trauma, infection, or tumor who were treated with the wound VAC with or without a silver negative pressure dressing between January 2003 and January 2010; the first 26 patients were treated with the wound VAC alone, and in the final 16 consecutively treated patients, the silver dressing was added to the regimen. We reviewed medical records to determine length of treatment as well as the number and type of surgical interventions these patients underwent. We compared the group treated with the wound VAC alone with those patients treated with the wound VAC and silver negative pressure dressing.

**Results:**

Hospital stay averaged 19 days in the VAC only group and 7.5 days in the VAC with silver dressing group (p < 0.041), length of overall treatment averaged 33 days in the VAC only group and 14.3 days in the VAC with silver dressing group (p < 0.022), number of operative débridements averaged 7.9 in the VAC alone group and 4.1 in the VAC with silver dressing group (p < 0.001), and success of wound closure without soft tissue transposition was 16 of 26 patients in the VAC alone group and three of 16 patients in the VAC with silver dressing group (p < 0.033).

**Conclusions:**

Based on the reduced length of care and the number of surgical procedures these patients with massive wounds of the pelvis and extremities underwent, we now use the silver negative pressure dressing in combination with the wound VAC as part of routine care of such patients. These results may be used as hypothesis-generating data for future randomized studies.

**Level of Evidence:**

Level III, therapeutic study. See Guidelines for Authors for a complete description of levels of evidence.

## Introduction

Massive pelvic and extremity soft tissue loss remains a complex and cumbersome problem. Infection, trauma, and tumors are common etiologies and may result in a prolonged course of treatment resulting from delayed healing, persistent drainage, pain, and other complications [1, 4, 16, 17, 20]. Dressing changes and the need for repeat surgical débridement often result in extensive hospital costs, increased pain, and deconditioning of patients. Soft tissue rotational and free flaps may be used; however, there is often harvest site morbidity, extensive operative time, and prolonged hospitalization [6, 19].

Vacuum-assisted closure (VAC) technology has been shown to be effective in the management of soft tissue loss from infections, vascular insufficiency, radiation-induced soft tissue necrosis, and traumatic disorders [1, 28, 29, 32]. Additionally, the advent of a portable VAC unit allows patients to mobilize earlier and expedites the return to maximal function. It has been shown that bacterial colonization can increase with wound VAC therapy, possibly resulting in delayed or impaired healing [25, 26, 33]. The use of a silver negative pressure dressing in conjunction with the VAC may inhibit the colonization of drug-resistant organisms and sustain early granulation leading to expedited healing [27, 29–31]. There is little evidence that bacteria develop resistance from continuous exposure to silver concentrate [18, 20–24]. Silver has been associated with reduced inflammation and modulation of matrix metalloproteinases in studies regarding the effects on burn patients [10, 15].

Silverlon™ (Cura, Chicago, IL, USA) is a highly concentrated negative pressure dressing that is a knitted fabric material that has been silver-plated by means of a proprietary autocatalytic chemical (reduction-oxidation) plating technique. This technique coats the entire surface of each individual fiber from which the dressing is made, resulting in a very large surface area for the release of ionic silver. Silverlon has been shown to reduce surgical site infection [8, 11]. This technology avoids the deposition of silver crystals in the wound and has not been shown to be cytotoxic or to cause skin discoloration. We are not aware of any prior studies comparing the wound VAC with and without a silver negative pressure dressing in the treatment of massive wounds of the pelvis and extremities. Accordingly, we sought to determine whether a silver negative pressure dressing used in conjunction with a wound VAC decreases (1) the length of acute hospital stay and overall length of treatment; (2) the number of surgical débridements the patients underwent as part of their care; and (3) the likelihood of wound closure without soft tissue transposition.

Importantly, we have studied the use of the VAC before [29]; five patients from that report are included in this report, with additional clinical followup, as part of the control group (the group treated with the wound VAC but without the silver negative pressure dressing).

## Patients and Methods

**Fig. 1:**
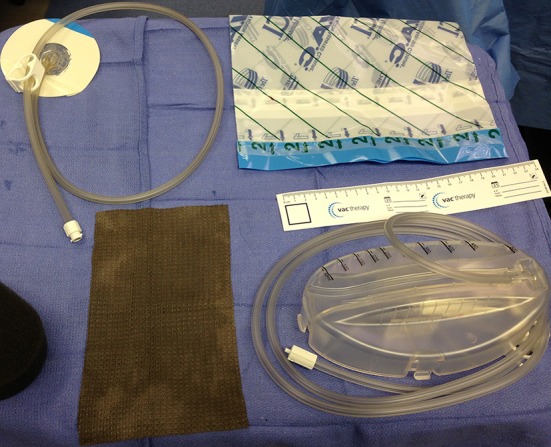
Basic wound VAC and silver negative pressure dressing setup is shown. The silver fabric dressing is placed between the VAC sponge and the wound. It is then sealed with an impervious sticky dressing.

Between January 2003 and January 2010, 42 patients were treated for massive pelvic and/or extremity wounds and were managed with the VAC device (KCI, San Antonio, TX, USA) by one surgeon (HJS). All patients with soft tissue defects > 200 cm^2^ involving the pelvis and/or the extremity that were treated with wound VAC therapy were included. The study group included 28 males and 14 females with an age range of 20 to 72 years (mean, 50 years). In this series, the first 26 patients were treated using the wound VAC alone, and in the last 16 consecutive patients, a Silverlon™ negative pressure dressing was used (Fig. 1).

The most common etiologies of soft tissue loss were infection (22), tumor (14), and trauma (six). Soft tissue infections were associated with a metal implant or prosthesis in 18 of 42 (43%) patients. All metal implants and/or prostheses were removed at the initial débridement. Antibiotic-impregnated spacers were placed during the wound management period. To date, six of the 18 patients with metal implants have undergone a second-stage reimplantation procedure. Twelve of 18 continue to have an antibiotic spacer in place. Eleven patients had a history of local radiation and 12 had a history of immunosuppression from either chemotherapy or organ transplantation. Twenty-two had surgical débridements before referral and 26 patients were on antibiotics before referral. The most common organisms cultured were sensitive *Staphylococcus aureus* (11), methicillin-resistant *S aureus* (nine), *Enterococcus faecalis* (eight), and *Staphylococcus epidermidis* (seven). Infectious disease consultation was obtained after the initial débridement once initial cultures were obtained.

Before initiation of VAC therapy, débridement of necrotic and/or infected tissue was performed in the operating room (OR) when indicated. In some instances, patients returned to the OR for serial débridements with VAC replacement. The VAC was changed at regular 2- to 3-day intervals for the wound VAC only group and every 7 days in the silver negative pressure dressing subgroup. The current recommendation for wound VAC management is to change the sponge no more than 72 hours because it can be difficult to remove as a result of overgrowth of exuberant granulation tissue. This is particularly important if the VAC dressing is to be performed as an outpatient, because removal after 72 hours may cause bleeding, pain, or retention of a portion of the sponge. A foul smell is also frequently appreciated with the VAC dressing, particularly if it is changed at intervals > 48 hours. The silver negative pressure dressing substantially slows the granulation ingrowth into the sponge and may be left on up to 7 days. The VAC dressings were either changed in the inpatient setting by a physician or as an outpatient by skilled nursing. The VAC change was performed under sterile technique in the OR if débridement was required. Otherwise it was changed in a clean but nonsterile environment either at the bedside or at the patient’s home. Patients were allowed to ambulate with a portable VAC unit and encouraged to do so. Wound measurements were recorded at each VAC change by either a physician or skilled nurse.

Treatment (whether with the wound VAC alone or with the wound VAC plus silver negative pressure dressing) continued until wound healing was accomplished by either primary or secondary intention, skin grafting, or soft tissue transposition. The minimum followup was 12 months (average, 35.3 months; range, 12–96 months).

**Fig. 2A–B:**
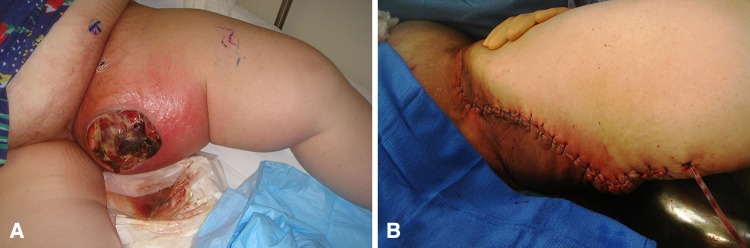
(**A**) Photograph of a large fungating mass involving the adductor compartment. The patient was treated with neoadjuvant chemotherapy and radiation followed by wide resection. A wound VAC with silver negative pressure dressing was applied for 3 weeks followed by primary closure. (**B**) Postoperative photograph showing primarily closed wound after 3 weeks of wound VAC treatment. The incision healed without further intervention.

The surgical technique for the silver dressing application is variable depending on the location and size of the wound. The dressing is a fabric material and easily cut into shapes to fit all wound geometries. It is recommended that the entire open wound be covered by the silver dressing as well as a portion of the surrounding skin. By overlaying the skin, it will protect it from the overlying VAC sponge. This will reduce skin irritation, breakdown, and maceration. The silver fabric dressing does not require fixation to the skin. The sponge is applied over the silver dressing and the application is completed by covering the sponge with an impervious sticky dressing. The wound VAC is then set to 125 mmHg on continuous mode. When removed the suction should be turned off or occluded and the dressing removed as one unit. Complex areas including the perineum, sacrum, and buttock were managed with VAC sponges secured in position with widely spaced circumferential staples and the impervious dressing was adhered to the skin with stoma paste (Convatec, Princeton, NJ, USA [29] (Fig. 2). Minor complications were reported in both VAC alone and silver dressing with VAC groups. As mentioned earlier, five patients were included in this study whose earlier results were published previously [29]. In this report, we extend followup on these five patients by a mean of 32 months (range, 16–45 months). All of these patients were treated in the wound VAC group without silver negative pressure dressings.

Institutional review board approval was obtained and patients gave consent for the use of their medical information for purposes of this study. The parameters evaluated included size of the soft tissue defect, duration of treatment, and patient compliance. Compliance was determined as per home nursing records and patient interview. Student’s t-test and log rank were used to determine statistical significance. Tests were considered significant if the p value was < 0.05.

## Results

The VAC with silver dressing group had shorter hospitalizations than the VAC only group (Table 1). The hospitalization for the VAC alone group was 19 days (range, 1–31 days) and for the VAC with silver negative pressure dressing 7.5 days (range, 2–22 days; p = 0.022). The average length of hospitalization for all patients was 8.1 days (range, 1–31 days).

**Table 1:** Comparison of patient outcomes undergoing wound VAC treatment with and without silver negative pressure dressing

Variable	VAC only [26]	VAC + silver dressing [16]	p value
Defect size (cm^2^; mean)	310.4 (200–611)	345.6 (220–500)	0.124
Immunosuppressed*	7 (26.9%)	5 (31.2%)	0.224
Radiation	8 (30.7%)	3 (18.8%)	0.073
Location			
Pelvis	14 (53.8%)	7 (43.8%)	0.612
Extremity	12 (46.2%)	9 (56.2%)	0.497
Surgical procedures	7.9 (3–12)	4.1 (2–9)	< 0.015
Treatment (days)	33.0 (5–91)	14.3 (7–30)	< 0.001
Average hospitalization (days)	19.1 (1–31)	7.5 (2–22)	< 0.033
Soft tissue flaps	16 (61.5%)	3 (18.8%)	< 0.024

Ranges shown in parentheses; * history of either chemotherapy or organ transplantation; VAC = vacuum-assisted closure.

The VAC with silver dressing group underwent fewer surgical débridements. The group treated with the VAC alone had 7.9 (range, 3–12) and the group with the VAC with silver negative pressure dressing had 4.1 (range, 2–9; p < 0.001). The average number of surgical débridements for all patients was 5.1 (range, 1–12).

**Fig. 3A–C:**
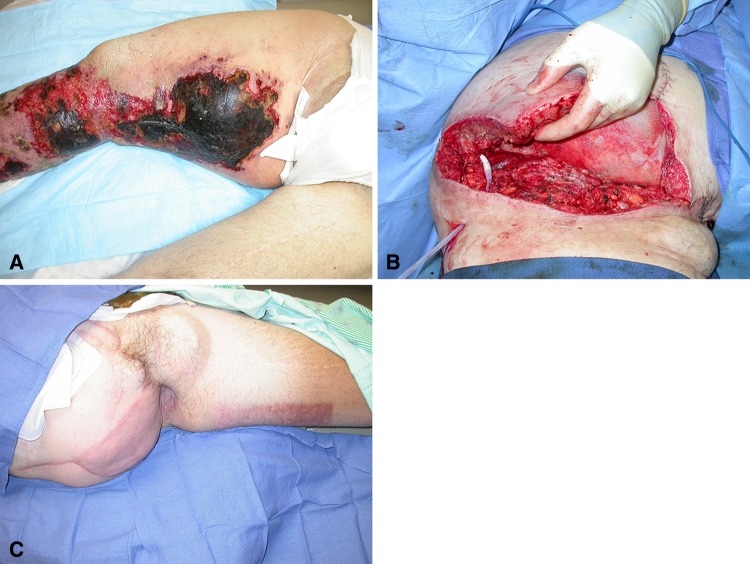
(**A**) Preoperative photograph of a patient with massive chondrosarcoma recurrence involving the pelvis, perineum, and thigh. Late ischemic/necrotic changes are seen. A hemipelvectomy with perineum/genital resection was performed. (**B**) Intraoperative photograph after resection. The massive wound was initially managed with a wound VAC with silver negative pressure dressing followed by skin grafting. (**C**) Postoperative photograph 8 weeks postoperatively from resection. A well-healed skin graft is shown.

Patients treated with the VAC plus silver underwent fewer soft tissue flaps for coverage (16 of 26 [62%] versus three of 16 [19%]; p = 0.024). Skin grafts were used in 11 of 26 (42.3%) patients in the VAC only group; nine of 11 (82%) healed without complication and nine of 16 (56.3%) patients in the silver group had skin grafts; all healed without complication. Twenty-two of 46 (47.8%) patients healed by secondary intention without the need for skin grafting (Fig. 3). Home health records indicated excellent compliance with only two of the 42 (4.7%) patients requesting discontinuation.

Since our previous publication [29], the five patients who were included in that earlier article have had a mean of 32 months additional followup (range, 16–45 months). These patients continue to have close followup for soft tissue sarcoma surveillance. None has been readmitted for wound complications and none has undergone further surgical procedures associated with wound complications.

## Discussion

The use of silver dressings has gained popularity in recent years [3, 5, 7, 9, 10]. There are several theoretical advantages including an antimicrobial inhibition and enhancement of soft tissue granulation [2, 12–14]. The prevalence of antibiotic-resistant organisms continues to rise; the antimicrobial effect of the local environment may be essential. Silver-resistant organisms have been reported but are extremely rare [27–29]. However, microbial resistance to silver remains somewhat controversial. It appears that silver negative pressure may be successfully used with a reduction of the frequency of dressing changes and reduction of a malodorous smell often reported with the use of wound VACs. The use of silver dressings has been substantiated as an adjunct in complex wounds [18, 20, 22], although it has not been studied in the setting of massive wounds such as those we evaluated. In this report we compared the use of the wound VAC with and without a silver negative pressure dressing in terms of length of care and the frequency with which surgical procedures were needed as part of that care.

This study had a number of limitations. First, because it was not a randomized controlled study, it is possible that some selection bias (among other kinds of bias) affected the decision to use the treatments under study. However, the fact that this was a sequential series should have offset some of the selection bias. It is important to note also that the patients had large soft tissue defects from different etiologies. However, they were comparably sized and all in compromised patients, and the etiologies did not vary over the course of the study. Related to that, wound characteristics (such as shape), adjuvant therapies, and host factors were not specifically evaluated or controlled; however, the study groups showed no significant differences in terms of immunosuppression, history of radiation, wound location, and size. Additionally, the study groups are relatively small; however, they were sufficiently large to allow us to detect significant differences between them. Finally, because this study involved a comparison of patients treated in two sequential series, the comparisons necessarily were historical. It is possible, if not likely, that other changes to treatments would have come into play during the time period in question, and it is possible, if not likely, that those cotreatments would have tended to inflate the apparent beneficial effects of silver dressings. Changes in hospital and outpatient care patterns likely also influenced issues such as duration of hospitalization over the period of time considered in this study.

To our knowledge, this is the only report of the use of the VAC in conjunction with a silver negative pressure dressing. The addition of a silver negative pressure dressing reduced the length of hospitalization compared with the VAC alone. The frequency in which the wound VAC is changed is reduced to weekly compared with two to three times per week. Additionally, the inhibition of granulation tissue into the sponge of the VAC appears to reduce the incidence of bleeding and pain that can be associated with the VAC sponge when used alone. Silver templated dressing technology has been mostly studied in chest, burns, and spine surgery with success in terms of reduced incidence of infection [8, 10, 11, 15]. These studies evaluated the topical application of this technology; however, the perforated, permeative dressing design that allows for the application of negative pressure by a VAC has not, to our knowledge, been reported before the present study.

The number of required surgical débridements used in the VAC with silver dressing likewise was reduced in our study. To our knowledge, there have not been previous reports specifically addressing this; however, the inhibition of bacterial colonization by the silver may be a contributing factor [2, 23, 27]. Surgical débridement of necrotic tissue remains an essential component of treatment. Reducing the frequency of VAC dressing changes to weekly in the VAC with silver dressing group may have protected the surgical wound from bacterial colonization during the hospitalization.

The use of wound VAC technology in conjunction with soft tissue transposition has been previously reported [16, 19, 29]. However, to our knowledge, there have not been prior reports of the use of silver dressings in conjunction with the wound VAC that address the use of soft tissue flaps. In our study, we observed a reduction in the use of soft tissue transposition in the VAC with silver dressing group. Only three of 16 patients (19%) in the VAC with silver dressing group required surgical intervention for soft tissue coverage compared with 16 of 26 (62%) in the VAC group without silver.

The VAC appears to facilitate soft tissue healing in patients with large, complex wounds. Patients should be prepared in advance that it may require a lengthy, cumbersome treatment; however, use of this tool appears to improve wound healing potential. The adjunct use of silver negative pressure appears to reduce the overall duration of care and decrease the likelihood that the patient will have other surgical procedures during treatment.
